# Lactate Suppresses Macrophage Pro-Inflammatory Response to LPS Stimulation by Inhibition of YAP and NF-κB Activation *via* GPR81-Mediated Signaling

**DOI:** 10.3389/fimmu.2020.587913

**Published:** 2020-10-06

**Authors:** Kun Yang, Jingjing Xu, Min Fan, Fei Tu, Xiaohui Wang, Tuanzhu Ha, David L. Williams, Chuanfu Li

**Affiliations:** ^1^ Department of Surgery, Quillen College of Medicine, East Tennessee State University, Johnson City, TN, United States; ^2^ Center of Excellence for Inflammation, Infectious Disease and Immunity, Quillen College of Medicine, East Tennessee State University, Johnson City, TN, United States

**Keywords:** lactate, yes associated protein (YAP), NF-kappa B, inflammatory cytokines, macrophages

## Abstract

Recent evidence from cancer research indicates that lactate exerts a suppressive effect on innate immune responses in cancer. This study investigated the mechanisms by which lactate suppresses macrophage pro-inflammatory responses. Macrophages [Raw 264.7 and bone marrow derived macrophages (BMDMs)] were treated with LPS in the presence or absence of lactate. Pro-inflammatory cytokines, NF-κB and YAP activation and nuclear translocation were examined. Our results show that lactate significantly attenuates LPS stimulated macrophage TNF-α and IL-6 production. Lactate also suppresses LPS stimulated macrophage NF-κB and YAP activation and nuclear translocation in macrophages. Interestingly, YAP activation and nuclear translocation are required for LPS stimulated macrophage NF-κB activation and TNFα production. Importantly, lactate suppressed YAP activation and nuclear translocation is mediated by GPR81 dependent AMKP and LATS activation which phosphorylates YAP, resulting in YAP inactivation. Finally, we demonstrated that LPS stimulation induces an interaction between YAP and NF-κB subunit p65, while lactate decreases the interaction of YAP and NF-κB, thus suppressing LPS induced pro-inflammatory cytokine production. Our study demonstrates that lactate exerts a previously unknown role in the suppression of macrophage pro-inflammatory cytokine production *via* GPR81 mediated YAP inactivation, resulting in disruption of YAP and NF-κB interaction and nuclear translocation in macrophages.

## Introduction

Recent studies have highlighted the role of aerobic glycolytic metabolism in the activation of innate immune cells (1). Generally, activated immune cells switch their metabolism from oxidative phosphorylation to aerobic glycolysis, resulting in increased production of lactate (2, 3). Historically, lactate has been considered a waste product of aerobic glycolysis (4). However, recent studies from cancer research have shown that tumor-derived lactate has immunosuppressive effects on immune cells (5), which are similar to those observed in the immunosuppressive phase of sepsis (6). Importantly, high serum lactate levels have been used to evaluate the severity and clinical outcome of septic patients (7–10). Thus, increased lactate concentration could significantly alter the function of immune cells during inflammation (11).

Recent evidence from cancer research suggests that lactate produced within the tumor microenvironment plays a role in regulating monocyte/macrophage pro-inflammatory response. Colegio *et al*. reported that cancer cell-derived lactate induces the M2-like polarization of cancer-associated macrophages, which contributes to an immunosuppressive microenvironment for cancer development (5). Selleri et al. have also shown that human mesenchymal stromal cell-secreted lactate induces a preferential differentiation of monocytes into M2 macrophages in a dose-dependent fashion (12). In addition, emerging studies showed that lactate delays LPS-induced signaling transduction (13) and decreases the production of pro-inflammatory cytokine TNFα (14). Mechanistic studies demonstrate that lactate signals *via* binding to its receptor G protein-couple receptor 81 (GPR81) (15). Indeed, either pharmacological inhibition of GPR81 by its antagonist or genetic deletion of GPR81 sufficiently attenuate the effects of lactate on immunomodulation (16, 17). Although previous studies indicate that lactate exerts a regulatory effect on immune cells *via* GPR81, the specific downstream mechanisms have not been completely elucidated.

Yes associated protein (YAP) is an important co-effector in the Hippo signaling pathway and it plays an important role in cell survival, proliferation and differentiation (18). Growing evidence suggests that YAP is highly involved in inflammation-related diseases, such as atherosclerosis (19) and pancreatitis (20). Importantly, Liu *et al*. reported that the Hippo signaling pathway in *Drosophila* participates in innate immune responses by regulating the production of antimicrobial peptides *via* the Toll like receptor (TLR) mediated pathway (21). Interestingly, Gao *et al*. have recently shown that TNFα promotes interaction between the NF-κB subunit p65 and YAP to synergistically regulate the transcription of hexokinase 2 in breast cancer cells (22). These data lead us to hypothesize that YAP and NF-κB could cooperatively regulate the functions of immune cells.

The present study investigated whether lactate could regulate YAP and NF-κB activation, thereby contributing to lactate-suppressed macrophage pro-inflammatory response. We demonstrated that lactate exerts suppressive effects on NF-κB and YAP activation *via* activation of AMPK and LATS in a GPR81-mediated signaling, resulting in reduced pro-inflammatory cytokine production in macrophages.

## Materials and Methods

### Animals

YAP^flox^ (JAX 030532), Lyz2-Cre (JAX 004781) and wildtype C57BL/6J (JAX 000664) mice were purchased from Jackson Laboratory (Indianapolis, IN). Macrophage specific YAP knockout mice were generated using the Cre/Loxp system. In general, *YAP* ^flox/flox^ mice were crossbred with lysozyme2 (Lyz2)-Cre mice. Genotypes for the macrophages specific deletion of YAP were confirmed by PCR analysis using the following primers: YAP F: 5′ CCC AAA TTT GAA TCA TTG GGG TCT TTG C 3′; YAP R1: AAC AAA ACC TGG GGA ACG ACT GGG CAC T 3′; YAP R2: GTG CAT AGC TGC ATA ACT TCG TAT AAT GT 3′. 410 bp for flox allele (F-R1), 315 bp for wild type allele (F-R1) and 230 bp floxed (deleted) allele (F-R2). Cre gene expression was also examined by PCR. The experiments outlined in this manuscript conform to the Guide for the Care and Use of Laboratory Animals published by the National Institutes of Health (NIH Publication, 8^th^ Edition, 2011). The experimental protocols were approved by the ETSU Committee on Animal Care.

### Cecal Ligation and Puncture (CLP) Polymicrobial Sepsis Model

Polymicrobial sepsis was induced by CLP as described previously (23). Briefly, mice (WT sham, N = 6; WT CLP, N = 6; WT sham lactate, N = 6; WT CLP lactate, N = 6; *YAP^-/-^* sham, N = 6; *YAP^-/-^* CLP, N = 6) were anesthetized by 5.0% isoflurane. After anesthesia, the abdomen was shaved, and the cecum was exposed through a 1 cm midline incision. The cecum was ligated between 3^rd^ and 4^th^ vascular arcade with a 4-0 silk suture and punctured with a 25-gauge needle. Sham surgically operated mice were served as sham controls. Following surgery, a single dose of resuscitative fluid was administrated by subcutaneous injection. Recovery was facilitated by placing mice on a heated pad. Lactic acid (pH 6.8, 0.5 g/kg body weight) was intraperitoneally injected 6 h after CLP or sham surgical operation. Terminal collection of blood was done *via* the vena cava. Serum was prepared as described elsewhere and stored at −80°C for further experiments.

### Preparation of Bone Marrow-Derived Macrophages (BMDMs)

Bone marrow stromal cells were isolated from femurs and tibias of C57BL/6 mice (N = 4) in media composed of EMEM-LG (Sigma, St., Louis, MO), 10% fetal calf serum (HyClone, Thermo Fisher Scientific Waltham, MA), glutamine 2 mM, and penicillin/streptomycin (50 U/ml and 50 mg/ml (Sigma) and supplemented with heparin at a final concentration of 5 U/ml (24). The isolated cells were washed twice with medium without heparin, plated in a petri dish at a density of 2 × 10^6^ cells/cm^2^ and incubated in culture medium containing M-CSF (20 ng/ml) at 37°C with 5% CO_2_ for seven days to induce bone marrow-derived macrophages (BMDMs). To confirm the macrophage population, the expression of macrophage marker, F4/80, was examined by Western blot.

### Preparation of Peritoneal Macrophages

Peritoneal macrophages were collected from both WT (N = 4) and *YAP^-/-^* (N = 4) mice as described previously (25). Briefly, inner skin lining the peritoneal cavity was exposed by cutting the outer skin of peritoneum. Five microliter PBS was injected into the peritoneal cavity using a G27 needle. To increase the yield of peritoneal macrophages, attached macrophages were dislodged by gently massaging the peritoneum. Fluid was collected and centrifuged 500*g* for 5 min. The purity of macrophage population was confirmed by flow cytometry with anti-CD11b and anti-F4/80 antibodies.

### *In Vitro* Experiments

To determine whether lactate could induce cellular injury, macrophages (RAW 264.7) were treated with different doses of lactate (0, 5, 10, 20 and 40 mM) for 6 h. The cells were harvested for flow cytometry analysis of cell apoptosis and cell death using a FITC Annexin V Apoptosis Detection Kit with PI (BioLegend, San Diego, CA).

To determine the suppressive effect of lactate on LPS stimulated responses of macrophages, RAW 264.7 cells were treated with lactate (10 mM) 1 h prior to lipopolysaccharide (LPS, 0.1 µg/ml) stimulation for 24 h. The supernatants were collected for the measurement of inflammatory cytokines with commercially available ELISA kits (PeproTech, Rocky Hill, NJ). In separate experiments, bone marrow-derived macrophages (BMDMs) were treated with lactate (20 mM) 1 h prior to LPS stimulation for 24 hrs. The supernatants were collected for the measurement of pro-inflammatory cytokines with commercially available kits (PeproTech, Rocky Hill, NJ).

To investigate the effect of lactate on LPS stimulated NF-κB activation, macrophages were treated with lactate (10 mM) 1 h prior to LPS (0.1 μg/ml) stimulation for 1 h. The cells were harvested for the isolation of the cytosolic and the nuclear proteins as described previously (24). In separate experiments, immunofluorescent staining with anti-NF-κB subunit p65 antibody (CST, Denver, MA) was performed to examine the nuclear translocation of p65.

To determine whether YAP is necessary for LPS stimulated NF-κB activation and pro-inflammatory cytokine production in macrophages, macrophages (BMDMs) were treated with the YAP inhibitor verteporfin (VP, 1 mmol/L) or transfected with siRNA-YAP (80 nmol/L) or siRNA-Control (80 nmol/L) (24), respectively prior to LPS stimulation.

To examine whether lactate-induced suppression of YAP activation will be mediated through GPR81 dependent AMPK activation, macrophages were treated with a GPR81 inhibitor (3-OBA) or AMPK inhibitor (Compound C) 1 h before lactate administration. After LPS stimulation for 1 h, the cells were harvested for the isolation of the cytosolic and the nuclear proteins as described previously (24) and the levels of GPR81, AMPK phosphorylation and YAP phosphorylation were examined as by Western blot.

To examine whether there was an interaction between YAP and NF-κB following LPS stimulation, cells were treated with lactate 1 h prior to LPS stimulation. The cells were harvested for the isolation of cellular proteins. Immunoprecipitation was performed (26) with anti-YAP antibody (CST, Denver, MA) followed by immunoblotting with anti-NF-κB p65 antibody (CST, Denver, MA). The immunoprecipitation was also performed with anti-NF-κB p65 (CST, Denver, MA) antibody followed by immunoblotting with anti-YAP antibody (CST, Denver, MA).

### ELISA

The levels of pro-inflammatory cytokines (TNFα, IL-6) were measured in the supernatants from cultured macrophages using commercially available ELISA kits (PeproTech, Rocky Hill, NJ) according to the instruction provided by the manufacturer.

### Immunoblotting

Immunoblotting was performed as described previously (23, 24, 26). Briefly, the cellular proteins were separated by SDS-polyacrylamide gel electrophoresis and transferred onto 0.45 NC nitrocellulose membranes (Amersham Pharmacia, Piscataway, NJ). The NC membranes were incubated with appropriate anti-YAP, anti-p65, anti-p-p65, anti-IκBα, anti-p-IκBα (CST, Danvers, MA), respectively, followed by incubation with peroxidase-conjugated second antibodies (CST, Danvers, MA). The membranes were incubated with the ECL system (ThermoFisher Scientific, Walttham, MA) and the signals were quantified using the G:Box gel imaging system by Syngene (Syngene, USA, Fredrick, MD).

### Flow Cytometry

Annexin V-propidium iodide (PI) staining was performed with an FITC-Annexin V Apoptosis Detection Kit with PI (BioLegend, San Diego, CA). Briefly, macrophages (Raw 264.7) was seeded in a 6-well plate (1 × 10^5^) and treated with different concentrations of lactate (0, 5, 10, 20, and 40 mM) for 6 h. Cells were detached and washed twice with cold BioLegend cell staining buffer, and then suspended in Annexin V binding buffer at a concentration of 5 × 10^6^ cells/ml. One hundred microliter (100 μl) of cell suspension was transferred to a flow tube, and the cells were incubated with FITC-Annexin V and PI for 15 min at room temperature in dark. For detection, 400 μl of Annexin V binding buffer was added to each tube, and the samples were analyzed on a Becton Dickinson FACSCalibur instrument (BD, US). FlowJo 7.6 software was used for data analysis.

### Immunostaining of NF-κB p65 Nuclear Translocation

Cells were grown on glass coverslips in 24-well plate and fixed in 4% paraformaldehyde for 15 min, permeabilized with 0.1% Triton X-100 in PBS in room temperature for 20 min, followed by 3% BSA blocking for 30 min. NF-κB p65 (CST, Danvers, MA) was diluted in 3% BSA/PBS at 1:00 and incubated overnight. The NF-κB p65 antibody was detected with anti-rabbit IgG secondary antibody Alexa Fluor 594 (ThermoFisher Scientific, Waltham, MA). The coverslips were mounted onto microscope slides in Vectashield mounting medium for fluorescence containing DAPI (Vector Laboratories, Burlingame, CA) and fluorescent images were visualized using Leica TCS II Confocal Microscope System.

### Immunoprecipitation

Approximately 200 µg of cellular proteins were isolated from BMDMs were subjected to immunoprecipitation with two µg of antibody against YAP or NF-κB subunit p65 (Santa Cruz Biotechnology, CA). After incubation overnight at 4°C, 15 µl of protein A-agarose beads (Santa Cruz Biotechnology) were added into the immunoprecipitation as previously described (26). The precipitates were washed for four times with lysis buffer before subjected to immunoblotting with the appropriate antibodies.

### Statistical Analysis

The data are expressed as mean ± SD. Comparisons of data between groups were made using one-way analysis of variance (ANOVA), and Tukey’s procedure for multiple-range tests was performed. Probability levels of 0.05 or smaller were used to indicate statistical significance.

## Results

### Lactate Attenuates LPS Stimulated Pro-Inflammatory Cytokine Production

**Figure 1:**
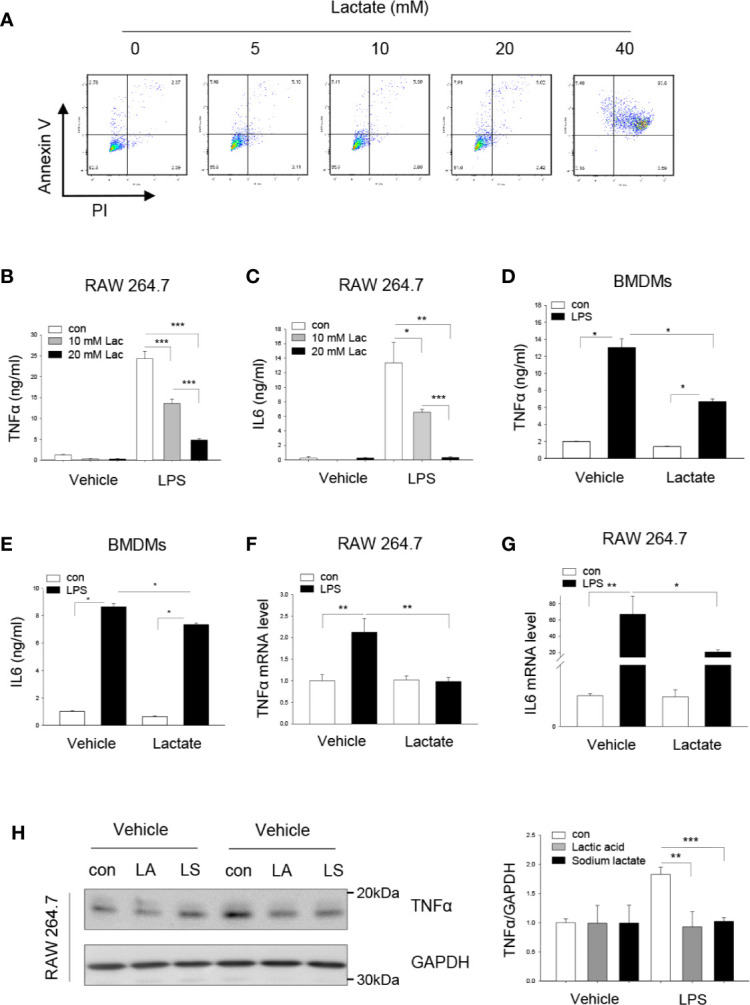
Lactate attenuates LPS stimulated macrophage TNFα and IL-6 production. **(A)** The effect of lactate concentration on macrophage apoptosis and death was determined by flow cytometry. Macrophages (Raw 264.7) were treated with lactate at 0, 5, 10, 20, and 40 mM for 6 h. Macrophage apoptosis and death were analyzed by flow cytometry. **(B–G)** Lactate (10 or 20 mM) attenuates LPS stimulated macrophage (Raw 264.7, B, C) and bone marrow derived macrophage (BMDM, D and E) TNFα and IL-6 production. Macrophages (RAW 264.7) were treated with lactate at 10 and 20 mM 1 h prior to LPS (0.1 μg/ml) stimulation for 24 h. The mRNA levels of TNFα and IL-6 were measured by qPCR **(F, G)**. **(H)** Acidic pH of lactic acid did not alter LPS stimulated macrophage TNFα production. Macrophages (Raw 264.7) were treated with lactic acid (LA), lactate that was adjusted pH with sodium hydrogen (LS) to the control pH level 1 h prior to LPS stimulation. Cellular TNFα levels were examined by Western blot. N/group = at least three independent experiments. Values are mean ± SD. *P* values were made by comparison with indicated groups (*P < 0.05; **P < 0.01; ***P < 0.001).

Recent studies have shown that lactate suppresses LPS stimulated pro-inflammatory cytokine production in monocytes/macrophages and mast cells (14, 27). However, the mechanisms are unknown. We first evaluated whether lactate at high concentration will induce macrophage apoptosis. Towards this end, macrophages (Raw 264.7) were treated with lactate (0, 5, 10, 20, and 40 mM) for 6 h and flow cytometry was performed to examine cell death and apoptosis. As shown in **Figure 1A**, lactate up to 20 mM did not induce macrophage apoptosis or cell death. However, treatment of macrophages with lactate at 40 mM caused significant cell apoptosis/death. In separate experiments, we treated macrophages with lactate at 10 or 20 mM concentration 1 h before the cells were stimulated with LPS for 24 h. Pro-inflammatory cytokines TNFα and IL-6 in the medium were measured with commercially available kits. **Figures 1B, C** show that lactate at 10 mM significantly decreased LPS-stimulated TNFα and IL-6 production in macrophages. Lactate at 20 mM resulted in even more attenuation of LPS-stimulated TNFα (**Figure 1B**) and IL-6 (**Figure 1C**) production. We also observed that treatment of bone marrow-derived macrophages (BMDMs) with lactate at 20 mM markedly suppressed LPS-stimulated TNFα and IL-6 production (**Figures 1D, E**). However, suppressive effect of lactate on LPS-stimulated TNFα production was greater than in IL-6 production in BMDMs. In addition, lactate also attenuated LPS-increased mRNA levels of TNFα (**Figure 1F**) and IL-6 (**Figure 1G**), indicating that lactate could regulate LPS stimulated TNFα and IL-6 mRNA expression. To rule out the possible effect of lactate acidic pH on LPS stimulated pro-inflammatory cytokine production, we adjusted lactate acidic pH in the medium to the equal levels of control and LPS groups and observed that adjusted lactate acidic pH to the control levels did not alter the suppressive effect of lactate on LPS-induced increases in TNFα protein levels (**Figure 1H**). These data suggest that lactate exerts suppressive effects on LPS stimulated pro-inflammatory cytokine production in macrophages.

### Lactate Attenuates LPS-Stimulated NF-κB Activation and Nuclear Translocation

**Figure 2:**
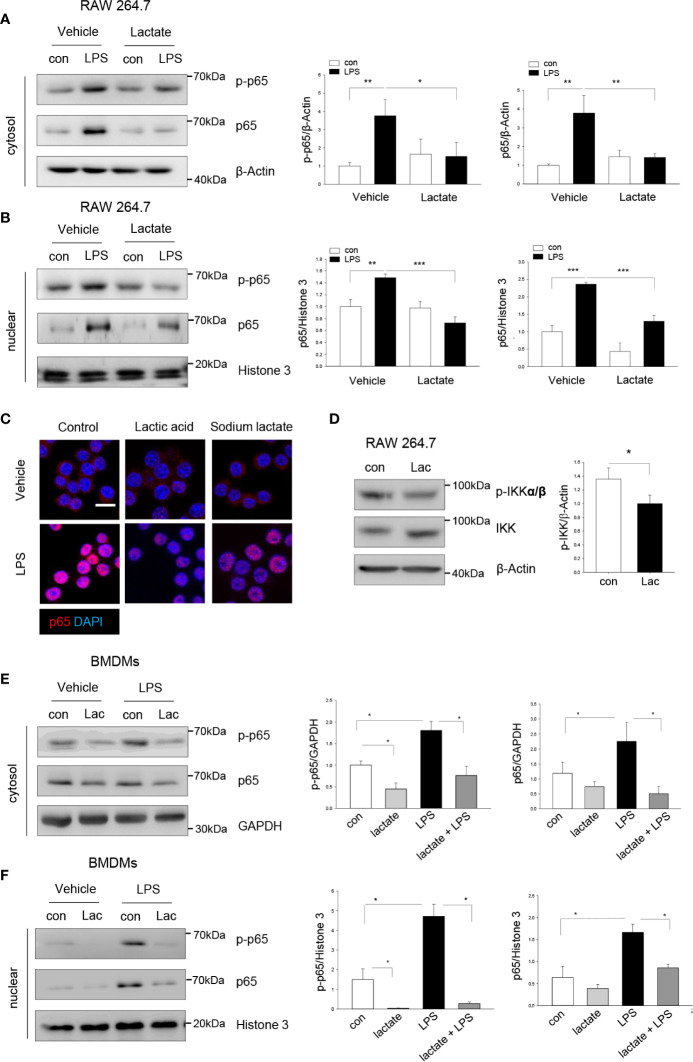
Lactate attenuates macrophage NF-κB activation and nuclear translocation. Macrophages (Raw 267.4) were treated with lactate 1 h prior to LPS stimulation for 1 h. The cells were harvested for the isolation of the cytosolic and nuclear proteins. **(A, B)** Lactate (10 mM) suppresses LPS increased levels of NF-κB subunit p65 and its phosphorylation in the cytosol **(A)** and nuclear translocation **(B)**. **(C)** Immunostaining shows that lactic acid (10 mM) or sodium lactate (10 mM) suppresses LPS stimulated NF-κB p65 (red) nuclear translocation. Nuclear was stained with DAPI (blue) (Scale bar, 10 µm). **(D)** Raw 264.7 cells were treated with lactic acid (10 mM) for 1 h and p-IKKα/β and total IKK were assayed by Western blot. **(E, F)** Lactate suppresses NF-κB activation and nuclear translocation in LPS treated bone marrow derived macrophages (BMDMs). BMDMs were treated with lactate 1 h before the cells were stimulated with LPS. The levels of NF-κB subunit p65 and its phosphorylation in the cytosol **(E)** and in the nucleus **(F)** were measured by western blot. N/group = at least three independent experiments. Values are mean ± SD. *P* values were made by comparison with indicated groups (*P < 0.05; **P < 0.01; ***P < 0.001).

To investigate the mechanisms by which lactate suppressed LPS-stimulated macrophage pro-inflammatory response, we first examined the effect of lactate on LPS-induced NF-κB activation. It is well known that LPS stimulates NF-κB activation which is an important transcription factor regulating pro-inflammatory cytokine production (28). Macrophages were treated with lactate 1 h prior to LPS stimulation. **Figures 2A, B** show that LPS stimulation significantly increased the levels of phosphorylated NF-κB subunit p65 in the cytosol (**A**) and the nucleus (**B)**. Immunofluorescent staining also showed that LPS stimulation increased p65 nuclear localization in macrophages. However, lactate treatment attenuated the LPS-induced NF-κB p65 phosphorylation and nuclear translocation (**Figures 2A–C)**. IκB kinases (IKKs) phosphorylation initiates phosphorylation and degradation of IκBs, which leads to the release of NF-κB dimers from NF-κB-IκB complex (29). It has been shown that pharmacological inhibition of IKKs reduced NF-κB p65 nuclear localization and pro-inflammatory cytokine production (30–32). Interestingly, we found that lactate suppressed phosphorylation of IKKα/β (**Figure 2D**), suggesting that lactate-reduced activation of NF-κB p65 could be achieved by IKK inactivation. Similarly, lactate suppressed LPS-induced NF-κB p65 phosphorylation (**Figure 2E**) and nuclear translocation (**Figure 2F**) in BMDMs. Together, these data suggest that lactate attenuated LPS-induced pro-inflammatory cytokine production is mediated, in part, by suppressing of NF-κB activation and nuclear translocation in macrophages.

### YAP Is Required for LPS-Stimulated NF-κB Activation and Nuclear Translocation

**Figure 3:**
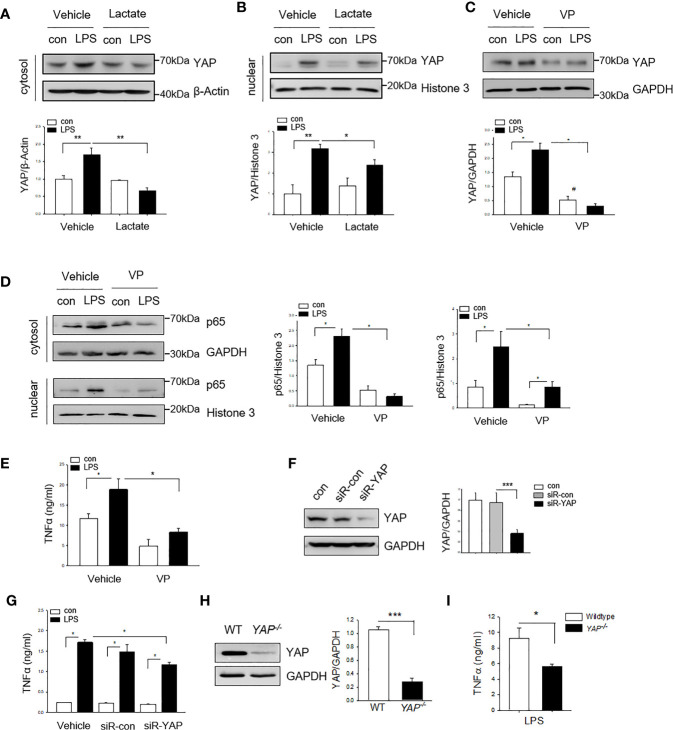
Lactate suppresses YAP expression and nuclear translocation in LPS treated macrophages. Macrophages were treated with lactate (10 mM) 1 h prior to LPS stimulation for 24 h. The cells were harvested for the isolation of the cytosolic and the nuclear proteins for western blot analysis of YAP levels. **(A, B)** Lactate suppresses LPS-stimulated increases in the levels of YAP in the cytosol **(A)** and in the nucleus **(B)**. **(C, E)** Inhibition of YAP with a YAP inhibitor (VP) suppresses LPS induced increases in the levels of YAP **(C)**, NF-κB subunit p65 in the cytosol **(D)** and the nucleus **(D)** as well as TNF-α production **(E)**. **(F, G)** Silencing of YAP by siRNA transfection suppresses YAP expression **(F)** and attenuates LPS-stimulated TNFα production **(G)**. **(H, I)** YAP deficiency suppresses LPS stimulated TNFα production in peritoneal macrophages. Peritoneal macrophages were isolated from WT and YAP specific macrophage knockout mice and stimulated with LPS. YAP expression was assayed by western blot **(I)**. TNFα production was measured with ELISA kit **(I)**. Values are mean ± SD. *P* values were made by comparison with indicated groups (*P < 0.05; **P < 0.01; ***P < 0.001).

YAP is an important co-effector in Hippo signaling and plays a critical role in cell proliferation and differentiation (18). Recent evidence has shown that YAP activation contributes to innate immune response (21). Next, we examined whether YAP is involved in lactate-suppressed NF-κB activation in macrophages. Macrophages were treated with lactate 1 h prior to LPS stimulation and YAP expression was assessed by western blot analysis. **Figures 3A, B** show that LPS stimulation increased the levels of YAP in the cytosol (**A**) and the nucleus (**B**). However, treatment of macrophages with lactate suppressed LPS-induced increases in the levels of YAP in the cytosol (**A**) and the nucleus (**B**). To investigate whether YAP is required for LPS stimulated NF-κB activation and pro-inflammatory cytokine production, we treated macrophages with a YAP inhibitor (Verteporfin, VP) 1 h prior to LPS stimulation and examined the levels of NF-κB subunit p65 in the cytosol and the nucleus. As shown in **Figures 3C, D**, LPS treatment increased the levels of YAP (**C**) and NF-κB subunit p65 (**D**), and promoted p65 nuclear localization (**D**). Interestingly, YAP inhibition prevented LPS-stimulated increases in the cytosolic and the nuclear NF-κB subunit p65 levels (**Figure 3D**). YAP inhibition also suppressed LPS-stimulated TNFα production in macrophage (**Figure 3E**). To confirm YAP is needed for LPS induced pro-inflammatory cytokine production in macrophages, we silenced YAP *via* transfection of YAP specific siRNA for 24 h prior to LPS challenge and observed that silencing of YAP significantly decreased YAP protein levels (**Figure 3F**) and suppressed LPS-stimulated TNFα production in BMDMs (**Figure 3G**). To further validate the role of YAP in LPS-induced pro-inflammatory cytokine production, we isolated peritoneal macrophages from both wild type (WT) and myeloid-specific YAP knockout mice (*YAP^-/-^*) and treated them with LPS. YAP deficiency (**Figure 3H**) markedly suppressed LPS-stimulated TNF-α production in peritoneal macrophages (**Figure 3I**). Collectively, these data suggest that YAP is an important co-effector for LPS-stimulated NF-κB activation and pro-inflammatory cytokine production in macrophages. In addition, these data indicate that lactate suppressed pro-inflammatory cytokine production can be mediated *via* regulation of YAP activation and nuclear translocation in macrophages.

### Lactate Promotes YAP Phosphorylation and Degradation in Macrophages

**Figure 4:**
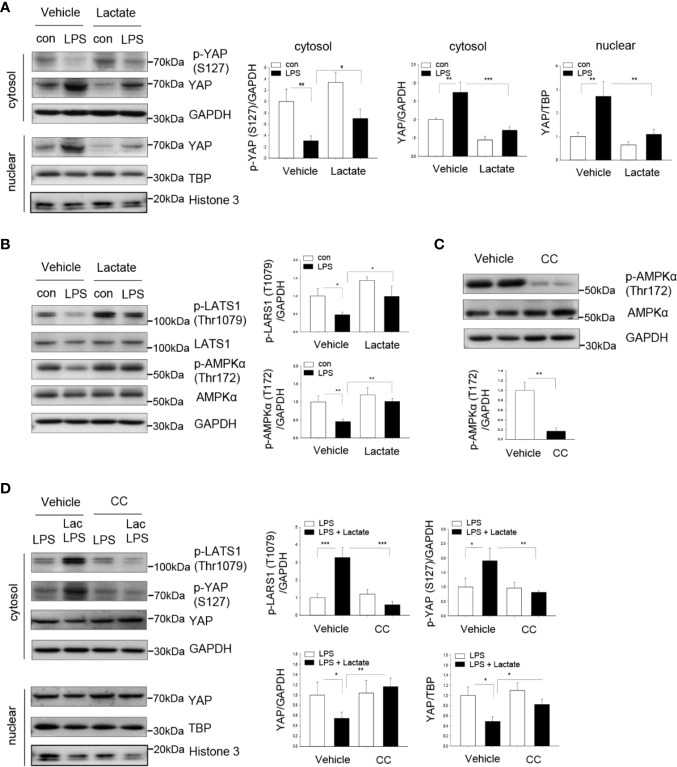
Lactate promotes YAP phosphorylation and decreases YAP nuclear levels in macrophages. Macrophages (Raw 267.4) were treated with lactate (10 mM) 1 h prior to LPS stimulation. **(A)** Lactate promotes YAP phosphorylation and suppresses LPS induced increases in YAP levels in cytosol and in the nucleus. **(B)** Lactate promotes LATS and AMPK phosphorylation. **(C, D)** AMPK inhibition by compound C (CC) abolished lactate promoted phosphorylation of LATS and YAP and attenuated lactate decreased YAP levels in the cytosol and in the nucleus. Macrophages were treated with AMPK inhibitor CC 1 h before the cells were treated with lactate followed by LPS stimulation. AMPK inhibitor decreased AMPK phosphorylation, prevented lactate-induced phosphorylation of LATS and YAP and abolished lactate decreased YAP levels in the cytosol and in the nucleus. N/group = at least three independent experiments. Values are mean ± SD. *P* values were made by comparison with indicated groups (*P < 0.05; **P < 0.01; ***P < 0.001).

We next investigated whether lactate-suppressed NF-κB activation and pro-inflammatory cytokine production could be mediated through inhibiting YAP activation and nuclear translocation. YAP phosphorylation at Ser127 results in YAP inactivation and degradation (33). We treated macrophages with lactate prior to LPS stimulation and examined YAP phosphorylation by western blot. **Figure 4A** show that LPS significantly increased the levels of YAP in the cytosol and the nucleus. However, lactate administration markedly increased YAP phosphorylation at Ser127 and decreased YAP levels in the cytosol and the nucleus (**Figure 4A**), when compared with LPS-treated cells. These data suggest that lactate induces YAP phosphorylation, thereby suppressing LPS-induced YAP activation and nuclear translocation in macrophages.

### Lactate Induces AMPK and LATS Phosphorylation

To investigate the mechanisms by which lactate induced YAP phosphorylation and inactivation, we examined the effect of lactate on AMPK activation. We have previously shown that AMPK activation resulted in YAP phosphorylation and inactivation (34). **Figure 4B** shows that treatment of macrophages with lactate significantly increased AMPK phosphorylation. Previous studies have demonstrated that LATS is a downstream molecule of AMPK and upstream of YAP (34, 35). In addition, LATS phosphorylation leads to YAP phosphorylation and inactivation (34, 35). Interestingly, **Figure 4B** shows that lactate treatment increased LATS phosphorylation, which may contribute to YAP phosphorylation and inactivation. To conform that AMPK activation resulted in LATS phosphorylation, we treated macrophages with AMPK inhibitor (Compound C) prior to lactate administration. As shown in **Figure 4C**, Compound C efficiently suppressed AMPK phosphorylation. Importantly, AMPK inhibition abolished lactate-induced phosphorylation of both LATS and YAP and attenuated lactate-suppressed YAP nuclear translocation (**Figure 4D**). Collectively, these data suggest that lactate-suppressed YAP activation is mediated through activation of AMPK, which results in phosphorylation of LATS, and finally leads to YAP phosphorylation and degradation.

### GPR81 Mediates the Suppressive Effect of Lactate on YAP and NF-κB Activation and Cytokine Production in Macrophages

**Figure 5:**
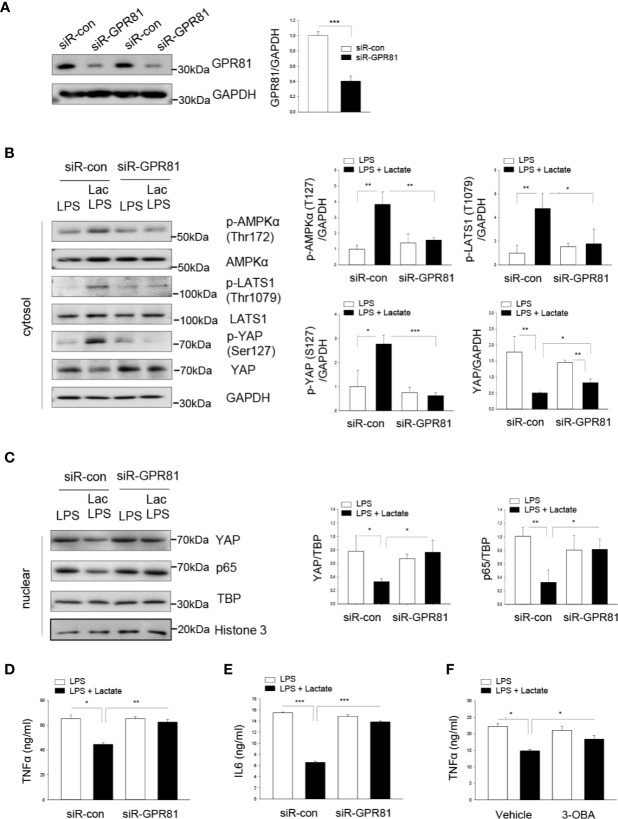
Lactate promotes the phosphorylation of AMPK, LATS1 and YAP through the GPR81 receptor. Macrophages were transfected siRNA for GPR81 or scrambled siRNA control 24 h before the cells were treated with lactate followed by LPS stimulation. **(A)** Knockdown of GPR81 expression was assayed by Western blot. **(B, C)** Silencing of GPR81 prevented lactate promoted phosphorylation of AMPK, LATS and YAP **(B)**, and attenuated lactate decreased nuclear YAP levels **(C)**. **(D, E)** Silencing of GPR81 attenuated lactate-decreased production of TNFα **(D)** and IL-6 **(E)** pro-inflammatory cytokines in LPS-stimulated RAW 264.7 cells. **(F)** Inhibition of GPR81 by its antagonist 3-OBA partially abolished lactate-suppressed TNFα production in LPS-stimulated RAW 264.7 cells. N/group = at least three independent experiments. Values are mean ± SD. *P* values were made by comparison with indicated groups (*P < 0.05; **P < 0.01; ***P < 0.001).

GPR81 has been identified as a receptor for lactate (36). We then investigated whether GPR81 could mediate the suppressive effect of lactate on YAP activation and nuclear translocation. Macrophages were transfected with siRNA specific for GPR81 or scrambled siRNA for 24 h before the cells were stimulated with LPS in the presence or absence of lactate. Our results show that silencing of GPR81 suppressed GPR81 protein levels (**Figure 5A**), abolished lactate-induced AMPK, LATS, and YAP phosphorylation (**Figure 5B**), and attenuated lactate-decreased the nuclear levels of YAP and NF-κB subunit p65, thus enhancing LPS-stimulated YAP and NF-κB nuclear translocation (**Figure 5C**). These data suggest that the suppressive effect of lactate on YAP activation and nuclear translocation is mediated by GPR81-dependent AMPK activation.

Next, we examined the pro-inflammatory cytokine production following knockdown of GPR81 in LPS-stimulated macrophages with or without lactate treatment. **Figures 5D, E** show that silencing of GPR81 expression abolished the suppressive effect of lactate on LPS-stimulated TNFα and IL-6 production in macrophages. Treatment of macrophages with a GPR81 inhibitor (3-OBA) prior to lactate administration also attenuated lactate-inhibited LPS-stimulated TNFα production in macrophages (**Figure 5F**). Together, our data suggest that GPR81 plays a role in mediating the suppressive effect of lactate on pro-inflammatory cytokine production in macrophages.

### Lactate Suppresses an Interaction Between YAP and NF-κB in LPS-Stimulated Macrophages

**Figure 6:**
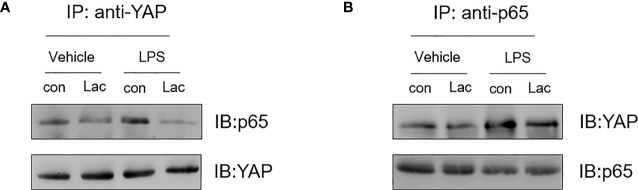
Lactate suppresses LPS induced an interaction between YAP and NF-κB subunit p65 in macrophages. Macrophages were treated with lactate 1 h before the cells were stimulated with LPS for 1 h. The cells were harvested for the isolation of cellular proteins. **(A)** Immunoprecipitations were performed with anti-YAP followed by immunoblot with anti-p65. **(B)** Immunoprecipitations were also performed with anti-p65 followed by immunoblot with anti-YAP.

The above data show that lactate inhibited YAP and NF-κB activation and the nuclear translocation, resulting in suppressing pro-inflammatory cytokine production in macrophages, indicating that YAP and NF-κB could cooperatively regulate pro-inflammatory cytokine production. To further define how YAP and NF-κB regulate pro-inflammatory cytokine production in LPS stimulated macrophages, we examined whether there is an interaction between YAP and NF-κB in macrophages following LPS stimulation. Macrophages were treated with lactate 1 h prior to LPS stimulation. Cellular proteins were harvested, and immunoprecipitations were performed with anti-YAP antibody followed by immunoblotting with anti-NF-κB subunit p65 antibody. As shown in **Figure 6A**, LPS stimulation induced an interaction of NF-κB p65 with YAP as evidenced by the presence of NF-κB p65 in the YAP immunoprecipitants. Similarly, LPS-induced interaction between YAP and NF-κB p65 was also evidenced by the increased YAP expression in anti-p65 immunoprecipitants (**Figure 6B**). However, lactate treatment markedly attenuated the LPS-induced interaction between YAP and NF-κB p65 (**Figures 6A, B**). These data suggest that LPS induced an interaction between YAP and NF-κB subunit p65 in macrophages and that lactate administration inhibited the interaction of YAP and NF-κB p65. This may be an important mechanism by which lactate attenuated LPS induced NF-κB activation and inflammatory cytokine production in macrophages.

## Discussion

The present study demonstrated that lactate exerts a suppressive effect on LPS-induced YAP and NF-κB activation and the nuclear translocation, resulting in inhibition of pro-inflammatory cytokine production in macrophages. Specifically, we found that lactate markedly attenuated LPS-induced NF-κB activation and nuclear translocation. Interestingly, we found that LPS stimulation also increased YAP expression and unclear translocation which is required for LPS-stimulated NF-κB activation and nuclear translocation. However, lactate induced YAP phosphorylation and inactivation, thus suppressing LPS stimulated NF-κB activation and nuclear translocation. We demonstrated that the suppressive effect of lactate on LPS stimulated NF-κB and YAP activation and nuclear translation is mediated *via* GPR81 receptor dependent AMPK activation.

Lactate is the end product of glycolytic metabolism (4). Historically, lactate has been considered as a waste metabolite or at best a biomarker in critical illness, including sepsis (10). However, recent growing evidence shows that lactate modulates immune responses in various cancers (5, 37, 38), indicating that lactate could be one of the critical molecules that link metabolism and immunity. Accumulation of lactate in the tissue microenvironment is also a feature of sepsis (10). It has been indicated that the physiological lactate concentration in blood or healthy tissues is approximately 1.5 – 3 mM (39), while the blood lactate levels in septic patients can reach over 20 mM (40). Remarkably, lactate levels can rise to 40 mM at the inflamed tissues (39). Previous studies using different doses of lactate, ranging from 10 to 100 mM, demonstrated that lactate promotes macrophage polarization towards an M2 anti-inflammation phenotype (5, 12) and suppresses pro-inflammatory cytokines TNFα and IL6 production (14, 27) through inhibiting NF-κB activation (13). Given that high lactate at high concentrations induces significant cell death and apoptosis in macrophages, we used 10- and 20-mM lactate in the current study. We observed that lactate decreased LPS-stimulated macrophage TNFα and IL-6 production in a dose dependent manner. To rule out the possibility of lactate acidification in regulating the response of macrophage to LPS stimulation, we adjusted the medium pH values to those of vehicle-administrated medium and found that both lactic acid and sodium lactate effectively suppressed LPS-induced TNFα expression in macrophages. Although we did not examine the effect of lactate on macrophage polarization toward an M2 phenotype, our data does indicate that lactate exerts a suppressive effect on the pro-inflammatory response of macrophages to LPS stimulation.

The transcription factor NF-κB regulates numbers of genes that involve in immune and inflammatory responses (41). p65 (RELA) is one of the subunits of NF-κB transcription factor family and functions predominately as a transcription activator of pro-inflammatory cytokines (42, 43). Posttranscriptional modification, such as phosphorylation, of p65 upon LPS stimulation dictates the canonical mechanism for its nuclear translocation and activation (44). To address the mechanisms by which lactate suppressed pro-inflammatory response in macrophages, we focused on whether lactate could regulate NF-κB activation in LPS treated macrophages. We found that lactate markedly suppressed LPS induced NF-κB activation and nuclear translocation which positively correlates with decreased pro-inflammatory cytokine production. In support of this observation, Hoque et al. reported that increasing concentrations of lactate reduced TLR4-mediated NF-κB activation in macrophages and monocytes (15). A previous study by Zhang et al. have shown that genetic deletion of cardiomyocytes-specific p65 reduced transcription of both TNFα and IL-6 (45). In agreement, we observed that lactate-decreased nuclear accumulation of p65 was accompanied by suppressed expression of TNFα and IL-6 mRNA levels.

YAP is an important downstream effector of the Hippo signaling pathway which plays a critical role in controlling cell proliferation and differentiation as well as organ size (18). A recent study has shown that in *Drosophila* the Hippo signaling pathway regulates innate immune response and is activated downstream of the Toll like receptor (TLR) pathway to regulate production of anti-microbial peptides (21). Emerging evidence has indicated that YAP is also implicated in the regulation of macrophage immune responses (46). Zhou *et al.* show that depletion of YAP in macrophages relieves pro-inflammatory responses in inflammatory bowel disease (IBD) *via* enhancing macrophage M2 polarization and suppressing IL6 production (47). Consistently, targeted inhibition of YAP suppressed the expression of IL6 and IL11 in endothelial cells (48).

We found in the present study that YAP is essential for LPS stimulated macrophage pro-inflammatory cytokine production. YAP inhibition by either siRNA or a YAP inhibitor significantly attenuated LPS-stimulated NF-κB nuclear translocation and activation, and reduced LPS-induced TNFα production, indicating that YAP may be a co-effector for NF-κB activation nuclear translocation. Indeed, we found that lactate treatment markedly decreased YAP nuclear translocation, which positively correlated with attenuation of NF-κB nuclear translocation and TNFα production. These data suggest that YAP is essential for maximal NF-κB activation and pro-inflammatory cytokine induction and that lactate attenuates LPS stimulated pro-inflammatory cytokine production *via* suppression of YAP activation and nuclear translocation.

To investigate how lactate suppressed LPS-stimulated YAP activation and nuclear translocation, we examined the effect of lactate on AMPK phosphorylation and activation. AMPK is a ubiquitously expressed kinase that acts as an energy sensor and regulates cellular metabolism (49). We have previously shown that AMPK phosphorylation resulted in YAP phosphorylation and inactivation (34, 35), indicating that AMPK is upstream of YAP activation (34, 35). Indeed, we found that lactate treatment significantly induced AMPK phosphorylation, leading to the phosphorylation of LATS1. Substantial studies, including our previous studies, have demonstrated that phosphorylation of LATS1 will result in YAP phosphorylation and subsequent degradation (34, 35). Activation of AMPK signaling has been reported to suppress YAP activation through phosphorylation of LATS1 (34, 35). To confirm that lactate-induced YAP phosphorylation and inactivation could be through phosphorylation of AMPK/LATS, we treated macrophages with AMPK inhibitor and observed that AMPK inhibition abolished the effect of lactate on YAP phosphorylation and inactivation. Our finding suggests that lactate suppresses YAP activation and nuclear translocation *via* activation of AMPK (34, 35).

G protein-coupled receptors (GPRs) are important cell-surface receptors that are intensively involved in signaling transduction in almost all cells, including macrophages (50). Numbers of studies have shown that GPRs induce the activation of AMPK, suggesting that AMPK serves as a downstream effector of GPR-mediated signaling (51–53). GPR81 has been identified as a receptor for lactate (36). Hoque *et al.* reported that lactate decreased TLR4-mediated NF-κB activation in macrophages and monocytes *via* a GPR81 dependent mechanism (15). Ranganathan et al. reported that GPR81 regulated intestinal homeostasis and protected mice from experimental colitis (16). Wang et al. have also shown that lactate promotes M2 polarization of macrophages in a GPR81-dependent manner (54). The current study demonstrated that lactate-suppressed pro-inflammatory response in macrophages is mediated through GRP81. We found that silencing GRP81 or inhibiting GRP81 by its antagonist abolished lactate-induced AMPK activation, which further attenuated YAP and NF-κB inactivation. Consistently, blockage of GPR81 signaling partially reversed lactate-reduced TNFα and IL6 production in LPS-stimulated macrophages.

Gao et al. reported that TNFα could increase an interaction between p65 and YAP in breast cancer cells (22). To address how YAP and NF-κB work together to regulate pro-inflammatory cytokine production in macrophages, we performed immunoprecipitations and demonstrated that YAP interacted with NF-κB subunit p65 to regulate macrophage inflammatory response to LPS stimulation and that lactate inhibited LPS-induced an interaction between YAP and NF-κB p65 in macrophages. Our finding revels a novel mechanism by which lactate attenuated LPS-stimulated NF-κB nuclear translocation *via* GPR81 mediated AMPK/LAST activation, leading to YAP phosphorylation and inactivation.

## Conclusions

Lactate exerts suppressive effect on TNFα and IL-6 production in LPS-stimulated macrophages. Mechanistic studies demonstrated that lactate suppressed LPS-stimulated NF-κB and YAP activation and nuclear translocation *via* its receptor GPR81-mediated AMPK/LATS activation.

## Data Availability Statement

The raw data supporting the conclusions of this article will be made available by the authors, without undue reservation.

## Ethics Statement

The experimental protocols were approved by the ETSU Committee on Animal Care.

## Author Contributions

KY: Designed, performed experiments, analyzed data and prepared manuscript. JX: Performed experiments and analyzed data. MF: Performed experiments and analyzed data. FT: Performed experiments. XW: Discussed experiments and the results. TH: Performed experiments. DW: Discussed data and prepared MS. CL: Designed experiments, discussed data and prepared MS. All authors contributed to the article and approved the submitted version.

## Funding

This work was supported, in part, by National Institutes of Health grants R01HL071837 (CL), R01HL153270 (CL), R01GM083016 (CL, DW), R01GM119197 (DW), and C06RR0306551 to ETSU.

## Conflict of Interest

The authors declare that the research was conducted in the absence of any commercial or financial relationships that could be construed as a potential conflict of interest.
